# A rare case of primary esophageal Paget’s disease with underlying invasive adenocarcinoma

**DOI:** 10.1055/a-2378-6256

**Published:** 2024-08-16

**Authors:** Xue Chen, Heng Zhang, Aihua Qian, Xi Chen

**Affiliations:** 1 66281 Department of Gastroenterology, Shanghai Jiao Tong University Medical School Affiliated Ruijin Hospital, Shanghai, China; 2 66281 Department of Pathology, Shanghai Jiao Tong University Medical School Affiliated Ruijin Hospital, Shanghai, China

A 53-year-old man presented to Ruijin Hospital, having been followed up for 9 months with an esophageal lesion that had initially been detected during a routine esophagogastroduodenoscopy (EGD) and had been diagnosed histopathologically as a high grade intraepithelial neoplasia (HGIN).

The inpatient EGD revealed a flat (0-IIb) and slightly reddish lesion spanning from 25 to 38 cm of the middle-to-lower esophagus ( Fig. 1 ). Magnifying endoscopy with blue-laser imaging (ME-BLI) identified an intrapapillary capillary loop (IPCL) pattern consistent with type B1 ( Fig. 1 **d–f** ). Notably, a 5 × 5-mm slightly elevated area with mild congestion was observed within the lesion at 26 cm ( Fig. 1 **a** ). The lesion remained unstained after the application of Lugolʼs solution and exhibited a partial pink-color sign from 30 to 33 cm of the esophagus, without any signs of deep invasion ( Fig. 1 **g–i** ). Pathology and immunohistology of the biopsy specimen revealed CK7+, CK19+, P53(missense mutation), P40(little+), P63(little+), CK5/6(partial+), P16(little+), Ki67(70%+), AE1/AE3+, CAM5.2+, CK20(−), SOX-10(−), villin(−), HER2(0), SATB2(−), GCDFP-15(−) ( Fig. 2 ), suggesting (i) extramammary Paget disease; (ii) invasive adenocarcinoma with Paget dissemination (M1 for the biopsy, more tissue would be needed for the evidence of invasive adenocarcinoma) 1 2 3 4 .

**Fig. 1:**
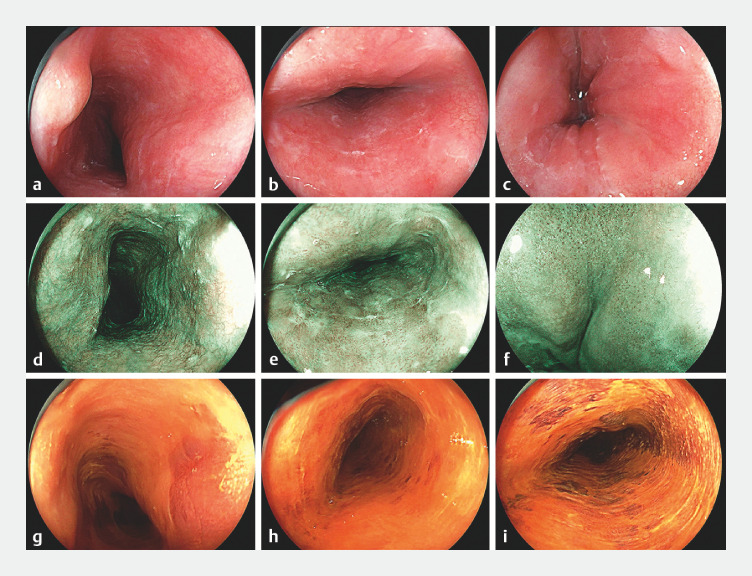
Endoscopic features of the lesion before radiofrequency ablation on: **a–c** white-light endoscopy; **d–f** magnifying endoscopy with blue-laser imaging; **g–i** after staining with Lugol’s solution.

**Fig. 2:**
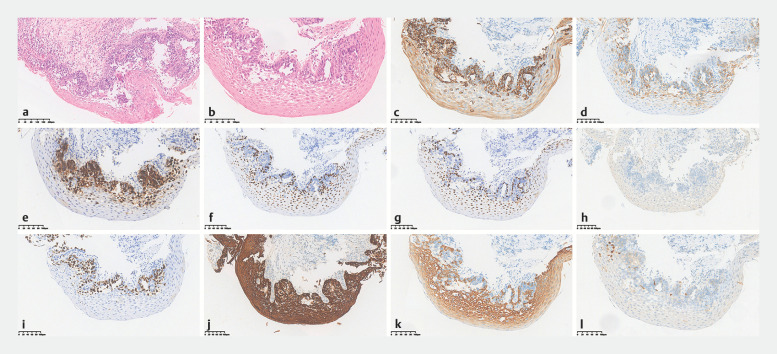
Microscopic appearance of the of biopsy specimen on: **a, b** hematoxylin and eosin (H&E) staining; **c–l** immunohistochemical staining with: **c** CK7(+); **d** CK19(+); **e** P53(missense mutation); **f** P63(little+); **g** P40(little+); **h** CK20(−); **i** Ki67(70%+); **j** AE1/AE3(+); **k** CK5/6(partial+); **l** P16(−).

Given the size of the lesion and the patientʼs refusal to undergo surgery, we performed radiofrequency ablation therapy ( Fig. 3 ). A follow-up EGD at 2 months post-treatment revealed persistent faintly red, rough mucosa extending from 26 to 38 cm of the esophagus ( Fig. 4 ). Notably, brownish areas were observed within the lesion under ME with narrow-band imaging (ME-NBI), with the majority of type B1 IPCL with partial type R vessels ( Fig. 4 **b, e** ). Subsequent Lugolʼs solution staining delineated an irregularly geographically distributed lesion with partial circumferential involvement ( Video 1 ). A biopsy taken at 30 cm demonstrated a pink-color sign ( Fig. 4 **f** ), confirming the previous diagnosis.

**Fig. 3:**
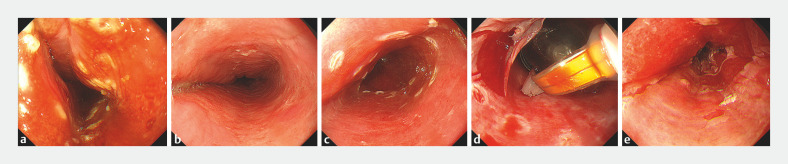
Endoscopic images during radiofrequency ablation therapy.

**Fig. 4:**
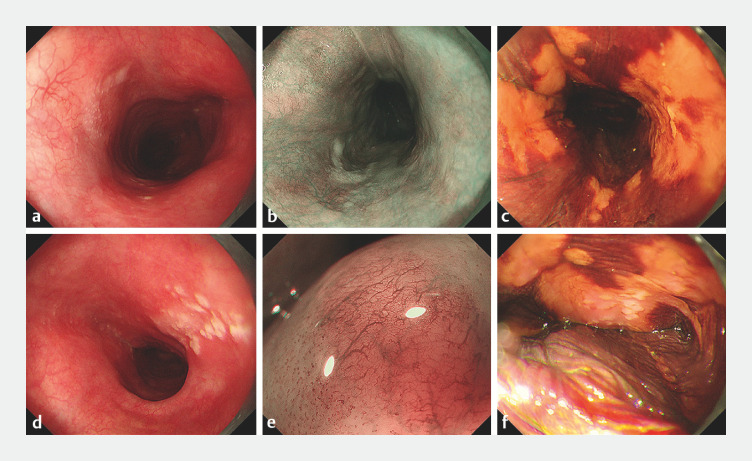
Endoscopic images during follow-up esophagogastroduodenoscopy 2 months after treatment on: **a, d** white-light imaging; **b, e** magnifying endoscopy with narrow-band imaging; **c, f** after staining with Lugol’s solution.

Features of a rare primary Paget’s disease of the esophagus under white-light endoscopy and magnifying endoscopy with blue-laser imaging/narrow-band imaging before and after radiofrequency ablation treatment.Video 1

The patient currently continues on regular EGD follow-up every 3 months at his local hospital. If the lesion were to worsen during follow-up, chemoradiotherapy would be considered.

Endoscopy_UCTN_Code_CCL_1AB_2AC

## Notes

Endoscopy E-Videos https://eref.thieme.de/e-videos
*E-Videos* is an open access online section of the journal *Endoscopy* , reporting on interesting cases and new techniques in gastroenterological endoscopy. All papers include a high-quality video and are published with a Creative Commons CC-BY license. Endoscopy E-Videos qualify for HINARI discounts and waivers and eligibility is automatically checked during the submission process. We grant 100% waivers to articles whose corresponding authors are based in Group A countries and 50% waivers to those who are based in Group B countries as classified by Research4Life (see: https://www.research4life.org/access/eligibility/ ). This section has its own submission website at https://mc.manuscriptcentral.com/e-videos .
